# A systematic review of the effect of dietary and nutritional interventions on the behaviours and mental health of prisoners

**DOI:** 10.1017/S0007114524000849

**Published:** 2024-07-14

**Authors:** Matthew Poulter, Shelly Coe, Catherine Anna-Marie Graham, Bethan Leach, Jonathan Tammam

**Affiliations:** 1 Centre for Nutrition and Health, Faculty of Health & Life Sciences, Oxford Brookes University, OX3 0BP, Oxford, UK; 2 Cereneo Foundation, Center for Interdisciplinary Research (CEFIR), 6354 Vitznau, Switzerland; 3 Lake Lucerne Institute AG, Rubistrasse 9, 6354 Vitznau, Switzerland; 4 Practice Plus Group, Hawker House 5-6 Napier Court, Napier Rd, Reading, Berkshire RG1 8BW, UK

**Keywords:** Prisoners, Nutrition, Mental health, Nutrition interventions, Deficiencies

## Abstract

Prisoners experience a higher burden of poor health, aggressive behaviours and worsening mental health than the general population. This systematic review aimed to identify research that used nutrition-based interventions in prisons, focusing on outcomes of mental health and behaviours. The systematic review was registered with Prospective Register of Systematic Reviews on 26 January 2022: CRD42022293370. Inclusion criteria comprised of current prisoners with no limit on time, location, age, sex or ethnicity. Only quantitative research in the English language was included. PubMed/Medline, Web of Science, EMBASE, PsycINFO and CINAHL were searched, retrieving 933 results, with 11 included for qualitative synthesis. Studies were checked for quality using the revised tool to assess risk of bias in randomised trials or risk of bias in non-randomised studies of interventions tool. Of the included studies, seven used nutritional supplements, three included diet changes, and one used education. Of the seven supplement-based studies, six included rule violations as an outcome, and only three demonstrated significant improvements. One study included mental health as an outcome; however, results did not reach significance. Of the three diet change studies, two investigated cognitive function as an outcome, with both reaching significance. Anxiety was included in one diet change study, which found a significant improvement through consuming oily fish. One study using diet education did not find a significant improvement in overall mental resilience. Overall, results are mixed, with the included studies presenting several limitations and heterogeneity. Future research should aim to consider increased homogeneity in research design, allowing for a higher quality of evidence to assess the role nutrition can play in improving the health of prisoners.

Malnutrition can significantly affect one’s ability to achieve optimal health and well-being^(1)^. Research has consistently identified the relationship between nutrition and health, demonstrating that inadequate nutrition could have detrimental effects at any stage of life^(2)^. From early fetal development, a pregnant mother’s dietary choices can play a pivotal role in preventing undernutrition in her child and influence the risk of obesity in the child’s adulthood^(3)^. Moving into adulthood, nutrition continues to play an important role in maintaining many aspects of health, where selected micronutrient deficiencies, such as a lack of vitamin D and Ca, could lead to an increased risk of osteoporosis^(4)^. These examples set a basis of evidence of deficiencies impacting health outcomes, though it is important to recognise that malnutrition does not solely refer to a lack of macro and micronutrients but also to overnutrition, such as a diet high in energy intake, fat, sugar and salt^(5)^. These are key health issues in many developed countries, where access to food is generally plentiful and is often overprocessed with fat, sugar and salt^(5)^. A diet high in these could increase the risk of obesity and obesity-related diseases such as diabetes, cancer and CVD^(4,6,7)^. While many of the identified effects of malnutrition relate to physical health and development, there is also emerging evidence of the impact of nutrition on mental health^(8)^.

Mental health is considered a state of mental well-being that enables people to cope with the daily stresses of life and contributes to their ability to work and engage with society and their community^(6)^. It is estimated that 970 million people worldwide live with at least one mental health disorder, with anxiety and depression being the most common^(2)^. Other prevalent mental health disorders include bipolar disorder, post-traumatic stress disorder, schizophrenia, eating disorders and disruptive behavioural disorders^(2)^. Health organisations and associations, including the National Health Service (NHS)^(9)^, the Association of UK Dietitians^(10)^ and the Royal College of Psychiatrists^(11)^, recommend a healthy diet as a crucial aspect of living with mental health disorders. This recommendation aims to aid recovery and ensure overall health while living with a disorder. Several reviews have identified the effects of diet on mental health outcomes, suggesting limited evidence of a relationship.

One such review identified that a Mediterranean diet, characterised by a high intake of fruits, vegetables, whole grains and fish, with low consumption of meat and dairy products, was inversely associated with the risk of depression^(12)^. However, this review noted caution with the results due to various methodological differences between the included studies. A later meta-analysis further identified the positive effects of a healthier diet on the risk of depression^(13)^. In addition, an unhealthier Western-style diet, characterised by a high intake of red meat, sweets and processed foods, is associated with a higher risk of depression. However, similar to the previous findings, there was heterogeneity among the studies included, and the authors stated that further research is needed to confirm these findings^(13)^. Nonetheless, these reviews provide a basis of evidence for a relationship between mental health and diet and the growing interest in this field. Yet, despite the body of evidence supporting nutrition’s role in human health, development and behaviour, select population groups remain vulnerable to malnutrition.

Vulnerable populations are defined as those who have limited resources to mitigate potential challenges in their lives, including challenges to their health outcomes^(14)^. Examples of vulnerable populations can include racial minorities, those living in poverty, the elderly, the homeless, people with mental health disorders and those in contact with the justice system, such as prisoners^(14,15)^.

Globally, the prison population is estimated to be approximately 10·8 million, though this figure could be as high as 11·5 million when accounting for incomplete data, as reported by the World Prison Population List^(16)^. Prisoners are considered a vulnerable population, often experiencing high levels of poor mental health and aggressive behaviours attributable to numerous factors. Examples include a life of poverty, past trauma, low educational achievements and poor general health^(17–19)^. The current population of UK prisoners stands at 88 126 as of 20 October 2023, with their mental health reported as highly compromised^(20)^. It has previously been reported that only approximately 10 % of prisoners receive treatment for mental disorders, while up to 70 % are estimated to suffer from at least one undiagnosed disorder^(18)^. The prison environment can be detrimental to health due to overcrowding, lack of purposeful activities (i.e. work/education) and inadequate nutritional intake^(17,21–23)^.

The WHO sets out that prisoners are entitled to basic human rights, including access to nutrition. However, evidence identifies that prisoners are either choosing a diet that fails to meet reference nutrient intakes or prison institutions that are not providing adequate options to meet nutritional recommendations. This issue has been identified in multiple countries^(17,23–27)^. We recognise that nutrition is not necessarily a solution to solve all health problems facing prisoners; however, good nutrition forms a key part of overall health and well-being. Research must aim at addressing nutritional needs, which have the potential to improve overall prisoner health, including mental health outcomes.

Therefore, this review aims to systematically examine dietary and nutritional interventions implemented in prisons among the prisoner populations to investigate their impact on mental health and a spectrum of behaviours. The term ‘behaviours’ is intentionally kept broad, encompassing a range of actions that may influence the overall well-being of prisoners. Notably, this includes behaviours linked to mental health, such as self-harm and aggression^(9,28,29)^. While presenting one of these behaviours alone is not enough to necessarily identify a mental disorder, in some cases, it can indicate an underlying undiagnosed mental disorder. Furthermore, disruptive behaviours are considered under the umbrella of mental health disorders, and the WHO notes that risk-taking behaviours such as substance abuse and violence can indicate underlying emotional issues^(2,6)^. This comprehensive approach in the review acknowledges the interconnectedness of mental health and behaviours, recognising both the prevalence of undiagnosed mental health concerns and the escalating levels of aggressive and violent behaviours observed across prisons globally^(30–35)^.

## Methods

This systematic review followed the Preferred Reporting Items for Systematic Reviews and Meta-Analyses guidelines 2020^(36)^. This review was registered in the International Prospective Register of Systematic Reviews (registration ID: CRD42022293370), where the study protocol and amendments can be found.

### Search strategy

Five electronic databases were searched: PubMed/Medline, Web of Science, EMBASE, PsycINFO and CINAHL, with no restriction on the publication date, using the filter for the English language. The initial search was conducted on 3 March 2022, with follow-up searches on 7 June 2022 and 18 October 2023 to ensure this review captured any new research published during the writing of this paper. The search strategy included key terms related to prison/prisoners, mental health, behaviours and diet interventions. Key terms were combined with Boolean operators ‘AND’ and ‘OR’. See Appendix 1 for full search terms. All retrieved results were organised in Microsoft Excel.

### Inclusion and exclusion criteria

Inclusion: (1) peer-reviewed articles published in English during any year globally; (2) study designs included are randomised controlled trials (RCT), cohort studies and case–control studies; (3) the study population must be prisoners at the time of the study, no restrictions on age, sex/gender, ethnicity or sexuality were applied; (4) any diet/nutrition-based intervention was considered; (5) any study where the outcome examined mental health or behaviours were included; and (6) studies must include a control or comparator group. Exclusion: (1) studies that have not been peer-reviewed and that are not in the English language, (2) qualitative and mixed method study designs, (3) populations that were not prisoners at any point during the study period, (4) non-diet-based interventions, (5) physical health outcomes and (6) studies lacking control or comparator.

### Data extraction

Data extraction began on 1 September 2022, with one reviewer extracting data, a second reviewer checking the work and any disagreements discussed. A third reviewer was consulted if necessary. Key data extracted included (1) general study characteristics: author(s), year, intervention type, location, group studied and study length; (2) characteristics of participants: number of participants, sex, age and inclusion/exclusion criteria; (3) intervention details: exposure, control/comparator, outcome domain and outcome measurement tools; and (4) results: key study results, including any effect measures (e.g. mean difference, OR), and overall conclusion.

### Quality assessment

Two reviewers independently evaluated the quality and risk of bias for the included studies. Risk of bias and quality assessment for randomised studies was conducted using the revised tool to assess the risk of bias in randomised trials^(37)^. The revised tool to assess risk of bias in randomised trials assesses bias based on five domains: the randomisation process, deviations from the intended intervention, missing outcome data, outcome measurement and selection of the reported result.

The risk of bias in non-randomised studies of interventions tool was used for non-randomised studies^(38)^. The risk of bias in non-randomised studies of interventions tool measures bias based on six domains: confounding, participant selection, intervention classification, deviations from intended intervention, outcome measures and results reported.

### Data synthesis

A meta-analysis was considered for this review; however, due to the anticipation of a diverse range of study types and methodologies, it was deemed to be of no benefit. Qualitative synthesis was conducted, where studies were grouped based on their intervention type and outcome. Additionally, due to the terms ‘prisoners/inmates/offenders’ being used interchangeably, for this review, only the term ‘prisoner/s’ will be used.

## Results

### Study selection

**Fig. 1.**
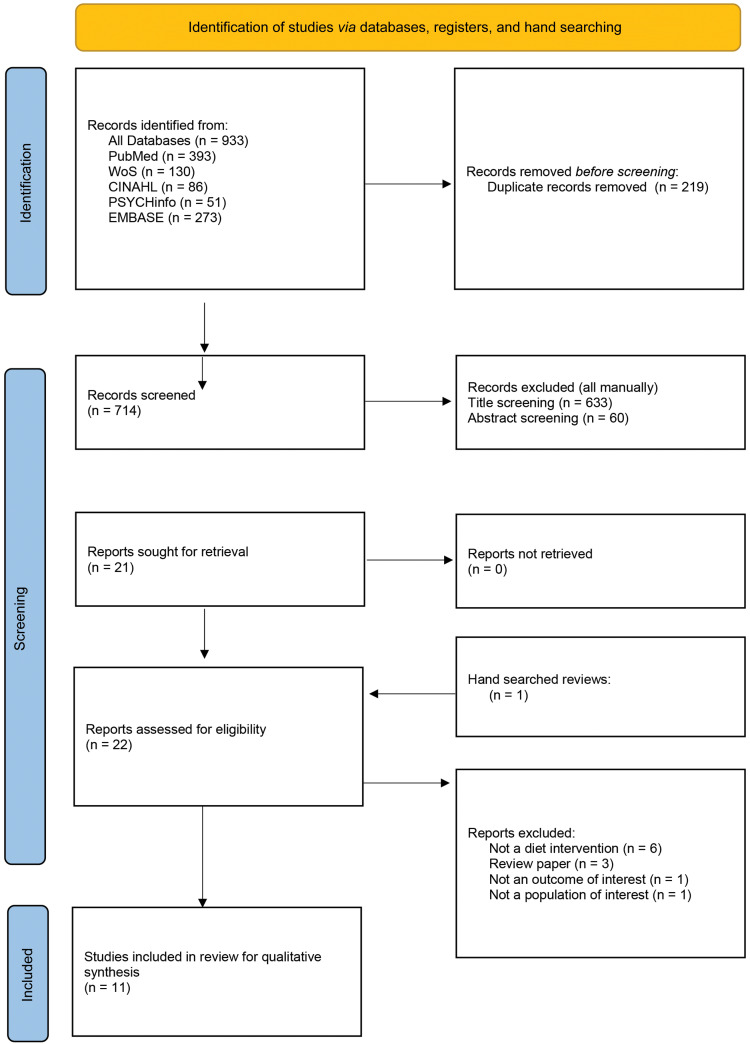
Preferred Reporting Items for Systematic Reviews and Meta-Analyses 2020 screening flow chart for the systematic review.

The database search identified 933 results, leaving 714 for title screening after duplicates were removed. After title screening, 633 records were removed with a further 60 removed following abstract screening. An additional paper was identified through hand-searching literature. The follow-up search on 18 October 2023 resulted in no further papers identified. In total, twenty-two results for full-text review were identified, after which eleven were deemed eligible for inclusion in qualitative synthesis; see Fig. 1 for the Preferred Reporting Items for Systematic Reviews and Meta-Analyses guidelines flow diagram^(36)^. See Table 1 for included study characteristics. See Appendix 2 for the excluded studies’ general characteristics and reasons for exclusion.

**Table 1.** Included study general and participant characteristics

Author, year	Intervention type	Study design	Location	Group studied, no. participants	Mean age (years, sd) (range)	Study length	Inclusion/exclusion criteria
Bachorowski *et al*. (1994)^(39)^	Diet change	RCT	USA	Juvenile male offenders, (*n* 48)	Mean not provided, (14–19)	3 d	Inclusion: Juvenile offenders, between 14 and 19 years old, with parental permission to volunteer for participation Exclusion: Those with medical records contraindicating participation, e.g. diabetes
Cortie *et al*. (2020)^(49)^	Supplements	RCT	Australia	Male adult prisoners, (*n* 131)	Intervention group: 33·7, 12·6 (19–80). Placebo group: 33·3, 10·3 (18–70)	16 weeks	Inclusion: Participants who were prisoners and 18+ years old
D’Asaro *et al*. (1975)^(40)^	Supplements	NRCT	USA	Inmates, (*n* 25)	N.D	8 weeks	Inclusion: Participants were expected to be incarcerated for 2 months minimum, and participation must be approved by the prison physicians and Warden
Gesch *et al*. (2002)^(48)^	Supplements	RCT	UK	Male adult prisoners, (*n* 231)	N.D	9 months	Inclusion: 18+ years old
Hansen *et al*. (2014)^(41)^	Diet change	RCT	USA	Male forensic inpatients, (*n* 95)	Intervention group: 42·76, 9·72 (21–60). Placebo group: 40·84, 9·14 (21–60)	23 weeks	Exclusion: IQ < 75
Hansen *et al*. (2015)^(42)^	Diet change	RCT	USA	Male forensic inpatients, (*n* 83)	Intervention group: 42·76, 9·72 (21–60). Placebo group: 40·84, 9·14 (21–60)	23 weeks	Exclusion: IQ < 75
Johnson *et al*. (2018)^(43)^	Diet education	Quasi Experimental Pilot	USA	Female prisoners, (*n* 29)	42·9, 12·0	12 weeks	Inclusion: 18+ years old, speaks and reads in English, incarcerated > 3 weeks but ≤ 12 months, ≥ 6 months to serve sentence, BMI ≥ 18 m/kg^2^ and received health clearance from the nurse practitioner (who was also the principal investigator)
Raine *et al*. (2020)^(47)^	Supplements	RCT	Singapore	Young male offenders, (*n* 145)	Intervention group: 19·22, 1·10. Placebo group: 19·29, 1·78. Control group 19·23, 1·31.	12 months	Inclusion criteria: 16+ years old, residing at the reformative training centre at the time of the study Exclusion criteria: Fish allergy, use of *n*-3 supplements within the past 3 months, intellectual disability and the ineligibility to enter the reformative training facility
Schoenthaler *et al*. (1997)^(44)^	Supplements	RCT	USA	Incarcerated Juveniles, (*n* 62)	Intervention group: 15·2 Placebo group: 15·2	3 months	Inclusion: Residents within the medical facility, with parental/guardian consent
Schoenthaler *et al*. (2021)^(45)^	Supplements	RCT	USA	Young male inmates, (*n* 449)	Low-dose group: 19·5, 1·4. High-dose group: 19·3, 1·5. Placebo group: 19·4, 1·4.	15 weeks	Inclusion: All prisoners 17–24 years old were eligible
Zaalberg *et al*. (2010)^(46)^	Supplements	RCT	The Netherlands	Male adult offenders, (*n* 221)	21, 1·5 (18–25)	3 months	No specific inclusion or exclusion criteria are given

RCT, randomised controlled trial; IQ, intelligence quotient; NRCT, non-randomised controlled trial; N.D, no data.

## Results

### Quality assessment

Of the eleven included studies, nine were assessed using the revised tool to assess risk of bias in randomised trials. Of these, eight were found to have low overall bias, while one was found to have some concerns (see Appendix 3). Two studies were assessed using the risk of bias in non-randomised studies of interventions tool, and both were found to have critical bias (see Appendix 4).

### General study characteristics

Of the eleven included studies, seven were conducted in the USA^(39–45)^. One study came from each of the Netherlands^(46)^, Singapore^(47)^, UK^(48)^ and Australia^(49)^. The earliest published study was from 1975^(40)^, with the next being in 1994^(39)^, and the remaining studies were all published after 2000. Study length varied, ranging from 3 d^(39)^ to 12 months^(47)^. Of the eleven studies, nine were RCT^(39,41,42,45–49)^ with the remaining two being non-randomised^(40,43)^.

### Participant characteristics

The number of participants in a study varied between 25 and 449. The supplementation intervention studies included the highest number of participants^(40,44–49)^. Only two studies included female participants^(43,44)^, one of which included both male and female participants^(44)^. The age of participants was not reported in two studies^(40,48)^, with one study only reporting the age range and not the mean age of participants^(39)^.

### Interventions, outcomes and results

The majority of studies, seven, were supplementation-based^(40,44–49)^, with only three assessing the impact of diet change^(39,41,42)^ and one investigating diet education^(43)^. See Table 2 for a summary of exposures, controls/comparators, outcomes and key results for the included studies.

**Table 2.** Included study exposure, control, outcomes and results summary

Ref.	Exposure(s)	Control/comparator	Outcome(s)	Outcome measurement tool(s)	Key results
(39)	Sucrose-loaded (78 g) breakfast, containing 636 kcal, 80 % carbohydrate, 11 % protein and 9 % fat	Comparator no-sucrose-loaded breakfast, containing 470 kcal, 62 % carbohydrate, 21 % protein and 17 % fat	(1) Behaviour	(1) 32-Behavioural Checklist (completed by experimenters)	There was an interaction between the nadir serum glucose group, order of breakfast and subscale of the behaviour checklist, indicating participants with low and normal nadirs were rated as having improved behaviours following the sucrose-loaded meal (*P* < 0·05).
(41)	Farmed Atlantic salmon for dinner (grilled or boiled), portion size 150–300 g, three times per week	No salmon, an alternative meal was provided, i.e. chicken, pork, beef, etc.	(1) Anxiety (biological) (2) Anxiety (self-report)	(1) HR, (2) rMSSD, (3) STAI	Regular fatty fish consumption improved HR, HRV baseline to post-intervention for those consuming the fish (*P* < 0·01; *d* = 0·45). State anxiety was also found to be reduced (*P* < 0·03; *d* = 0·43), though trait anxiety was found not to be reduced (*P* > 0·05).
(42)	Farmed Atlantic salmon for dinner (grilled or boiled), portion size 150–300 g, three times per week	No salmon, an alternative meal was provided i.e. chicken, pork, beef, etc.	(1) Cognitive function	(1) IGT, (2) ToH	Regular fatty fish consumption may improve executive functions in forensic inpatients with antisocial traits and a history of substance abuse (*P* = 0·013; *d* = 0·45).
(43)	Health education, and intervention monitoring/support with the nurse practitioner	No control	(1) Resilience	(1) Wagnild and Young’s Resilience Scale	Health education slightly improved resilience scores from baseline (148·50) to post-intervention (150·50), though not significantly (*P* = 0·437).
(49)	*n*-3 Capsules containing fish oil (32 % EPA and DHA) and a multivitamin supplement	Placebo and multivitamin	(1) Rule violations (2) Aggression (self-report)	(1) IBOS, (2) IRM, (3) AQ, (4) BADDS	Supplements were found to reduce aggression and rule violations, though not significantly (*P* > 0·05). Those found aggressive at baseline had a greater reduction in aggression and rule violations, though NS (*P* > 0·05).
(40)	Vitamin supplements	Placebo and diet education	(1) Abnormal behaviours	(1) Experiential World Inventory, (2) EPQ (neuroticism dimension), (3) Groesbeck Expanded, (4) Inmate Questionnaire	The effects of the vitamin supplements were found to be NS for all measures apart from four out of eight dimensions on the Experimental World Inventory, for the dimensions of sensory, time, ideation and self, indicating a decrease in feelings of depression. No level of significance was provided.
(48)	Vitamin, mineral, and fatty acid supplements	Placebo	(1) Rule violations	(1) Governor/minor reports	A reduction of 26 % (95 % CI 8·3, 44; *P* = 0·03) was seen for rule violations, indicating a reduction in antisocial behaviours in the supplement group.
(47)	*n*-3 Supplement 200 ml fruit-based drink, containing 300 mg DHA, 300 mg EPA, 180 mg α-linolenic acid, 60 mg DPA, 5 μg vitamin D and antioxidants (unspecified quantities)	200 ml fruit-based drinking containing 5 μg vitamin D and antioxidants (unspecified quantities) Control group: no drink	(1) Antisocial/aggressive behaviours, (2) rule violations	(1) AQ (self-report), (2) YPI (self-report), (3) RPQ (self-report and officer-report), (4) ASR (self-report), (5) CODDS (self-report and officer-report), (6) APSD (self-report and officer-report), (7) ABCL (officer-report), (8) SDAS (officer-report), (9) institutional infractions	Supplements were found to reduce antisocial behaviour, both short and long term, compared with control. After the intervention period (*P* = 0·006; *d* = 0·56), and 6 months post-intervention (*P* = 0·045; *d* = 0·43), based on the AQ self-reported measure. Officer-reported infractions were found not significantly different between groups (*P* > 0·36).
(44)	Vitamin-mineral supplements containing twelve vitamins (300 % US RDA) and eleven minerals (100 % US RDA)	Placebo	(1) Rule violation	(1) Rule violation reports	Those taking supplements were seen to experience an 83 % decrease in non-violent rule violations, compared with the 49 % seen in the placebo group (*P* = 0·008). Violent rule violations fell 80 % in the supplement group and 56 % in the placebo group (*P* = 0·044). Total rule violations fell 83 % for the supplement group and 55 % in the placebo group (95 % CI 15, 41; *P* = 0·005).
(45)	Intervention 1: Low-dose vitamin-mineral supplement containing 100 % of the recommended daily allowance, by US standards, of vitamins and minerals Intervention group 2: High-dose supplements containing the same amount of vitamins A, D, E, K, folic acid, biotin, Cu, iodine, Fe and Zn as the low-dose supplement, but had higher doses of the water-soluble B and C vitamins, and addition of Se, Cr, Mn and Mo	Placebo	(1) Rule violation, (2) mood, (3) personality traits, (4) intelligence	(1) Rule violation reports, (2) POMS, (3) EPQ	Supplements covering 100 % RDA were found to be able to reduce rule violations, compared with the placebo group (RR = 0·61, 95 % CI: 41, 90, *P* = 0·01). There was no significant result found between the placebo and high-dose group (RR = 1·48, 95 % CI: 0·99, 2·23, *P* = 0·06).
(46)	Mineral-vitamin supplement, *n*-3 fatty acids (EPA and DHA) and *n*-6 fatty acid g-linolenic acid	Placebo	(1) Aggression, (2) rule violations, (3) psychopathy	(1) AQ, (2) SDAS, (3) GHQ-28, (4) SCL-90	Supplements were found to reduce rule violations, (IRR = 0·60; *P* = 0·017). No effect was seen with aggression or psychopathy (*P* > 0·05).

HR, heart rate, HR; rMSSD, root mean square of successive differences between normal heartbeats; STAI, State-Trait Anxiety Index; HRV, heart rate variability; IGT, Iowa gambling task; ToH, tower of Hanoi; IBOS, Inmate Behavioural Observation Scale; IRM institutional reprimand misconduct; AQ, aggression questionnaire; BADDS, Brown’s attention-deficit disorder scale; EPQ, Eysenck personality questionnaire; AQ, Buss Perry Aggression Questionnaire; YPI, youth psychopathic trait inventory; RPQ, reactive-proactive aggression questionnaire; ASR, adult self-report; CODDS, conduct and oppositional defiant disorder scales; APSD, antisocial process screening device; ABCL, adult behaviour checklist; SDAS, social dysfunction and aggression scale; POMS, profile of mood states; RR, rate ratio; GHQ-28, general health questionnaire 28; SCL-90, symptom checklist-90; IRR, incident rate ratio.

### Effect of supplements on behaviours

Six supplement-based studies investigated behavioural outcomes, including rule violations and aggression. Four studies demonstrated a significant reduction in rule violations^(44–46,48)^, and only one study found a significant reduction in aggression^(47)^. Information on the types of supplements used in each study can be found in Table 2 and study characteristics in Table 1.

In a randomised placebo-controlled trial, Gesch *et al.* ^(48)^ found that the active supplementation group experienced a 26·3 % reduction in the number of infringements compared with the placebo group (*P* = 0·03). A similar RCT by Zaalberg *et al.* ^(46)^ conducted in the Netherlands also found that the number of recorded incidents of rule-breaking post-intervention was significantly less in the supplement group (incident rate ratio = 0·6; *P* = 0·017). Schoenthaler *et al.* ^(44)^ found in their RCT that for non-violent rule violations, there was an 83 % decrease in the experimental group compared with 49 % in the placebo group (*P* = 0·008). Violent rule violations fell 80 % in the experimental group and 56 % in the placebo group (*P* = 0·44)^(44)^. Overall, total rule violations fell 83 % for the supplement group and 55 % in the placebo group (95 % CI 15, 41; *P* = 0·005). A more recent RCT by Schoenthaler *et al.* ^(45)^ used high and low-dose multivitamin supplements, which found a statistically significant difference between the placebo and low-dose supplement group for serious rule violations (RR = 0·61; 95 % CI 41, 90; *P* = 0·01). The result between the placebo and high-dose group was not statistically significant (RR = 1·48; 95 % CI 0·99, 2·23; *P* = 0·06)^(45)^. Two of the more recent studies found no significant results for rule violations^(47,49)^.

Three of the supplementation studies investigated aggressive/antisocial behaviours^(46,47,47)^. Raine *et al.* ^(47)^ conducted an RCT resulting in the *n*-3 supplement group showing significantly lower self-reported antisocial behaviours compared with the placebo group after the 3-month experimental period (*P* = 0·006; *d* = 0·56). At 3 months post-intervention, the *n*-3 supplement group continued to report lower levels of antisocial behaviour compared with the placebo group, though the result failed to reach significance (*P* = 0·059; *d* = 0·39)^(47)^. At 6 months post-intervention, however, the *n*-3 group reported significantly lower levels of antisocial behaviours compared with the placebo group(*P* = 0·045; *d* = 0·43)^(47)^. No difference was found between groups based on officer reports of institutional infractions (*P* = 0·36)^(47)^. Cortie *et al.* ^(49)^ in a feasibility pilot RCT found that aggressive/antisocial behaviours were not significantly different through supplementation when assessing behaviours using the Inmate Behaviour Observation Scale (X^2^(1) = 0·83; *P* = 0·36), the self-report aggression questionnaire (X^2^(1) = 0·03; *P* = 0·82) and Brown’s attention-deficit disorder scale (X^2^(1) = 0·06; *P* = 0·64). A subset analysis was also conducted with participants who were characterised as aggressive based on their Inmate Behaviour Observation Scale baseline scores. Only eleven participants from the supplement group and eighteen from the placebo were eligible for this subset analysis. In the supplement group, there was an improvement of 7 % for the Inmate Behaviour Observation Scale (*P* = 0·566), 25 % for records of misconduct (*P* = 0·103), 19 % for aggression questionnaire (*P* = 0·371) and 2 % for Brown’s attention-deficit disorder scale (*P* = 0·910); however, all failed to reach significance. Zaalberg *et al.* ^(46)^ found that aggression, as measured by the staff-reported social dysfunction and aggression scale, demonstrated no significant difference between the supplement and placebo group (*P* = 0·23).

### Effect of supplements on mental health

Two of the supplementation studies included outcomes about mental health, with mixed results^(40,46)^. D’Asaro *et al.* ^(40)^ conducted a non-randomised controlled trial, finding that supplements did decrease feelings of anxiety; however, the significance was not reported. Zaalberg *et al.* ^(46)^ found, through the General Health Questionnaire-28, that there was a general trend for the supplementation group to experience better well-being; however, this did not reach significance (*P* = 0·069).

### Effect of diet change on mental health

Only one study which used diet change investigated mental health outcomes, where measures of anxiety improved^(41)^. Hansen *et al.* ^(41)^ aimed to identify the effects of diet change on mental health using an RCT. Heart rate and heart rate variability, measured as the root mean square of successive differences (rMSSD), were used as biological indicators of anxiety. The State-Trait Anxiety Inventory was used for self-reported anxiety. A diet change for the experimental group, which involved the provision of salmon three times per week for 23 weeks, resulted in a reduced heart rate for the experimental group from baseline to post-intervention (*P* < 0·01; *d* = 0·45). rMSSD increased in the experimental group from baseline to post-intervention, indicating anxiety improved (*P* < 0·01; *d* = 0·45). Additionally, vitamin D status was significantly related to rMSSD post-intervention (*P* < 0·05; *r* = 0·27), though not at baseline (*P* > 0·05). The State-Trait Anxiety Inventory revealed there was a significant decrease in state anxiety (*P* < 0·03; *d* = 0·43) for the experimental group, but no significant result was identified for trait anxiety (*P* > 0·05).

### Effect of diet change on cognitive function

One study investigated the effects of diet change on cognitive function, which found that diet change improved cognitive function^(42)^. Hansen *et al.* ^(42)^ conducted an RCT that was a part of the same diet change study by Hansen *et al.* ^(41)^, which involved the provision of fatty fish three times a week to the experimental group. Cognitive function was measured by the Tower of Hanoi task, measuring executive function (i.e. the ability to plan and solve tasks), and the Iowa gambling task, measuring risky decision-making. For the Tower of Hanoi task, the experimental group improved from baseline to post-intervention (*P* = 0·016; *d* = 0·35). A subgroup analysis was conducted with participants who had a previous history of drug/alcohol abuse, though no significant improvements in either group for the Iowa gambling task were found (*P* = 0·109; *d* = 0·41). The subgroup analysis for the Tower of Hanoi task showed that only those in the experimental group with a history of previous drug/alcohol abuse improved from baseline to post-intervention (*P* = 0·013; *d* = 0·45). Correlations between the nutritional status of EPA, DHA and vitamin D and cognitive performance were also conducted. No correlation between vitamin D status and cognitive performance was found. The effect of the sum of EPA and DHA on the sub-task with low working memory load task was found at baseline (*P* = 0·001; *r* = 0·41), though not at post-intervention (*P* = 0·858; *r* = 0·02).

### Effect of diet change on behaviour

One study investigated the effects of diet change on various behavioural outcomes including aggression and impulsivity, resulting in improved behaviours^(39)^. Bachorowski *et al.* ^(39)^ used diet change to identify the effects of sucrose on neuropsychological test performance, in three groups of juvenile offenders based on their serum glucose nadirs (low, borderline and normal). Participants ingested a sucrose-loaded breakfast and a no-sucrose control breakfast before a battery of neuropsychological tests, in a double-blind randomised crossover design. Participants were also observed by the researchers, who used a thirty-two-item behaviour checklist to evaluate observations of participants regarding their attention and motivation. The checklist was completed by the researchers after each day of neuropsychological tests and was formed from six subscales looking at restlessness, distraction, aggression, impulsivity, awkwardness and insecurity. It was found that there was an interaction between the nadir serum glucose group, order of breakfast and subscale of the behaviour checklist (*P* < 0·05). The results indicated that participants with low and normal nadirs were rated as having better behaviour following the sucrose-loaded meal, though overall, the study suggests the relationship between nadir serum glucose levels, breakfast type and behaviour improvement is complex.

### Effect of diet education on mental health

One study used diet education as the intervention, which investigated whether the mental resilience of participants would increase following the education course; however, no significant improvements were found^(43)^. Johnson *et al.* ^(43)^ conducted health education classes, comprising three sessions over 3 months each lasting between 60 and 90 min, as part of a quasi-experimental pilot, which focused on dietary education, that is, making healthier food choices, portion control, etc. The results showed a slight improvement in mental resilience between baseline, 6 weeks and 12 weeks. However, these improvements did not reach significance (*P* = 0·437).

## Discussion

### Overview of the results

The aim of this review was to identify existing literature, which reports the effects of nutritional interventions on the mental health and behaviours of prisoners. A previous systematic review was identified; however, this only looked at the effects of supplements on aggression outcomes^(50)^. Our review differed by considering all types of nutritional interventions on all mental health and behaviour outcomes. From the eleven studies included, three types of nutritional interventions appeared: diet change, diet education and dietary supplements.

### Dietary supplements

Supplementation studies were the most prevalent type of intervention in prisons, likely due to the ease of administering the intervention to a large population, in contrast to the more logistically and time-intensive methods of education and diet change. Although supplements were found to have significant positive results, there was a degree of heterogeneity across the studies’ overall conclusions.

Regarding the types of supplements used, three studies employed a combination of vitamin-mineral and fatty acid supplements^(46,48,49)^. Raine *et al.* ^(47)^ focused solely on the use of a fatty acid *n*-3 supplement. Two studies, D’Asaro, Groesbeck and Nigro^(40)^ and Schoenthaler *et al.* ^(44)^ utilised only a vitamin-mineral supplement. Schoenthaler *et al.* ^(45)^ offered either a low-dose vitamin-mineral supplement meeting 100 % of the RDA or a high-dose supplement exceeding the RDA for certain vitamins. D’Asaro, Groesbeck and Nigro^(40)^ provided only vitamin-mineral supplements to correct existing deficiencies within the sample population. Refer to Table 2 for a summary of the interventions used in each study, including supplement details.

#### Dietary supplements, rule violations and aggression

Rule violations emerged as the predominant outcome measured, with six out of the seven supplement studies incorporating this parameter. This preference is likely attributed to the accessibility of such data, as prison staff are generally mandated to report all rule violations, encompassing both violent and non-violent incidents, such as prisoner refusal to work. The evidence that shows the impact of supplements in reducing rule violations is largely positive. However, it’s noteworthy that four studies reported significant effects with similar medium effect sizes, emphasising the need for larger sample sizes to substantiate these findings^(44–46,48)^.

A key issue with the supplementation studies was identifying why any improvement in the outcomes measured may have occurred. For example, why rule violations may have reduced over the course of supplementation. This uncertainty is largely due to the variability between the supplements used in the studies. For example, Zaalberg *et al.* ^(46)^ replicated the study by Gesch *et al.* ^(48)^, yet the quantities of vitamins, minerals and fatty acids provided differed significantly in many cases. Both studies investigated *n*-3 fatty acids, yet Zaalberg *et al.* ^(46)^ provided almost five times as much *n*-3 fatty acids as Gesch *et al.* ^(48)^ and half the amount of vitamin D. As previously discussed, the evidence implicating omega fatty acids is limited when regarding mental health outcomes. However, given that rule violations can be linked more towards feelings of aggression and impulsivity, there is a potential that the underlying effects of omega deficiencies could explain the improvement seen. Previous research has identified some implications towards omega fatty acids regarding this, both in prison populations and children^(51,52)^. The two studies^(47,49)^ included in this review which identified no significant reductions from omega fatty acid supplements may have additional confounders leading to non-significant results.

The first of these two studies by Raine *et al.* ^(47)^ conducted their study in Singapore, with participants mostly being of Chinese or Malaysian descent. Non-significant findings could relate to the fact that Southeast Asian cuisine tends to contain higher levels of fish and, in turn, omega fatty acids when compared with Western diets^(47,53)^. Therefore, at baseline, this population would be less likely to have *n*-3 deficiencies, and supplementation would offer little benefit. However, there is no baseline dietary intake data provided, a common theme in the included literature. Therefore, it cannot be stated with certainty that the participants were *n*-3 sufficient at baseline. Furthermore, cultural factors are reported, where Singaporean institutions report fewer serious rule violations, particularly relating to drugs and alcohol, in comparison with studies in the Western world^(31)^. Therefore, it is conceivable that a less violent population would be less likely to demonstrate the potential effects of *n*-3 in reducing violent or antisocial behaviours. Though a decrease in rule violations was not observed, Raine *et al.* ^(47)^ demonstrated that self-reported aggression was reduced. Similarly, Cortie *et al.* ^(49)^ did not find results that meet significance for rule violations or self-report aggression, though there is a trend for rule violations to decrease in the supplement group. However, it is not possible to fully test the accuracy of this result due to a small sample size (*n* 131), with a *post hoc* power calculation finding that a sample size of *n* 600 would be needed to detect any significant effects.

Two studies that observed a reduction in rule violations used only vitamin-mineral supplements^(44,45)^. This leads us to consider whether the effects seen across the supplementation studies were attributed to a mechanism involving the vitamin and minerals component. Vitamin D is notorious for being deficient in populations globally, and in the context of a prison, this deficiency is more likely due to limited access to sunlight^(25,54,55)^. Among the included studies, Gesch *et al.* ^(48)^ found that vitamin D was deficient based on the baseline dietary assessment, with prisoners, on average, having an intake of 3·5 μg, while the current dietary recommendations suggest 10 μg per d^(56)^. Limited evidence suggests vitamin D’s role in improving behaviours from studies involving animals and children, though further research is necessary^(57,58)^. It is interesting to note that all the supplementation studies discussed in this review included some amount of vitamin D. The effects of supplements identified in this review could be attributed to any number of vitamins, minerals and fatty acids. This further highlights the common theme that the studies in this review lacked baseline dietary assessment, though this serves as an avenue for future research.

#### Dietary supplements and mental health

Mental health outcomes were not a prominent feature in the included studies. Only one study included general well-being as an outcome measure, where despite a general trend of improvement, this ultimately did not reach significance^(46)^. However, this study suffered from a high dropout rate, reducing the sample size and potentially limiting the significance of the result.

The earliest study included in this review, from 1975, found a significant decrease in self-reported anxiety; however, no significant value was provided^(40)^. Due to the lack of data and unclear methodology reported, this study was challenging to interpret and, thus, overall was found to be of high bias. High bias was in part due to the authors not reporting whether the outcome measures were suitable for the population. This study also had the smallest number of participants among the supplementation studies in this review, with a particularly small control group (*n* 7) compared with the experimental group (*n* 21), an issue pointed out by the authors. Participants in this study reported several side effects, including excessive hunger and skin eruptions. It is unknown if these were due to supplements or other reasons; however, no other study included in this review reported similar issues. Bias in the study was also discussed by the authors, noting that all participants were in the education programme which detailed the study and its purpose, potentially creating a placebo effect and subject bias^(40)^.

Despite the high burden of mental health disorders within prisons, there was very little consideration of mental health outcomes in the included studies. Previous research outside of the prison population identified beneficial effects of supplementation on mental health outcomes, particularly for vitamin D and depression, and recent guidelines recommend the use of *n*-3 for major depressive disorder^(59–62)^. Based on the limited evidence identified in this review regarding supplements and mental health, we conclude that research in this area is warranted.

### Diet change

Two diet change studies resulted from a larger study on mental health and nutrition^(41,42)^. Salmon was used primarily as a natural source of *n*-3 fatty acids and vitamin D, considered more beneficial than supplement use by the authors^(42)^. Both of these studies provided significant positive associations with fatty fish consumption, where the experimental group experienced reduced biological indicators of anxiety, state anxiety, reduced impulsivity, risky decision-making and improved planning and solving skills. Overall, results need to be interpreted with caution due to the nature of this type of dietary intervention, as issues lie in the inability to blind subjects. The authors reported this as a limitation and stated that both groups could have been influenced by the meals served, potentially perceiving salmon or chicken control as the healthier option.

Previous research outside the prison population has been unable to demonstrate a significant effect of fatty fish intake on rMSSD and heart rate variability^(63)^. Furthermore, a meta-analysis examining the effects of *n*-3 and polyunsaturated fats on anxiety and depression symptoms found little evidence to suggest a significant effect for preventing or reducing depressive or anxiety symptoms^(64)^. This meta-analysis highlighted a major limitation of the studies included, as many lacked a baseline dietary intake analysis – a limitation shared by the two diet change studies in this review. However, blood samples to monitor *n*-3 blood levels were taken to identify whether these had improved post-intervention for the experimental group. A more recent paper updating clinical guidelines about treating psychiatric disorders with nutritional therapies did recommend the adjunctive use of *n*-3 for those with major depressive disorder^(59)^. This recommendation is based on the results of five RCT and a statistically significant meta-analysis. Without understanding the overall baseline dietary status of participants, it is challenging to deduce what may be causing the significant effects, given that salmon provides more than *n*-3, including vitamin D, Se, and iodine, and is a source of protein. Whether the underlying biological mechanism was down to one or more of these other nutrients is unclear, though further investigation is warranted. Vitamin D was identified as a potential cause for improvements, as the fatty fish used in this study provided approximately 5 μg vitamin D, half the dietary recommendation for vitamin D. Positive associations were found between vitamin D status and rMSSD, indicating a potential role for vitamin D in improving anxiety. However, the authors reported that neither the control nor the supplement group was vitamin D deficient at baseline, requiring further investigation^(41,42,65,66)^.

The third diet change study included in this review differed from the two previous studies discussed, as Bachorowski *et al.* ^(39)^ aimed to identify the effects of sucrose on juvenile prisoners. This study did include an outcome focusing on behaviours; however, it was secondary to other outcomes which focused on neuropsychological tests not relevant to this review. Therefore, only the results of the behavioural checklist will be discussed. The authors had previously identified evidence that suggested an association between sugar consumption and behaviour, though the results were mixed^(67,68)^. Additionally, a meta-analysis on this topic identified that overall, there was little evidence supporting the implication of sugar on behaviour^(69)^. Bachorowski *et al.* ^(39)^ demonstrated a small significant effect of sucrose on behaviour, though these results should be accepted with caution. This diet change does not reflect real-life sugar consumption and focuses on brief short-term ingestion of sucrose in a controlled environment. In addition, this study had a small sample size and was found to have concerns in the revised tool to assess risk of bias in randomised trials. The results highlight that short-term sucrose ingestion produces beneficial results in juvenile prisoners. These include a reduction in distractedness and impulsivity seen across all three groups. Where the population of this study was prisoners, this could be seen as a desirable outcome. However, this study only considered short-term effects, and future work would benefit from identifying the effects of sugar consumption over a longer period. More recent research in this area has further highlighted the short-term benefits of sugar consumption for juveniles, believed to be down to the mechanism of the juvenile brain requiring more glucose than an adult^(70,71)^.

### Dietary education

While the only study to use nutrition education did not yield significant results, it did find a general increase in mental resilience^(43)^. The researchers noted that many participants at baseline were already reported to have high levels of resilience, potentially limiting the degree to which an individual could experience an increase. Overall, this study was found to have a high bias due to the lack of control for confounders, the absence of a valid measurement tool and the lack of a control group, which would aid in the validity of any results gained^(72)^.

Previous research on using education to improve mental health outcomes has yielded positive results, although it is challenging to make direct comparisons with this study due to differences in the measured mental health outcomes. For instance, a study focusing on nutrition education for obese women (*n* 44) in Japan, emphasising gut microbiota education, significantly improved self-measured levels of depressive symptoms^(73)^. Another study implemented education among a population of children in China (*n* 171), aiming to enhance self-measures of anxiety, and reported significant improvements^(74)^. These previous studies, however, featured larger sample sizes than the study by Johnson *et al.*, as well as broader exclusion criteria to reduce the risk of confounding. Nevertheless, these studies demonstrate that education can be a powerful tool for improving mental health outcomes.

The author acknowledged this study as a pilot study and the first to examine resilience in a prison population. Therefore, there are numerous opportunities for future studies to build upon, including the utilisation of high resilience levels and current mental health treatment as exclusion criteria, employing a larger population size and incorporating a control group for a more comprehensive before-and-after comparison^(75,76)^.

### Limitations

The research studies included in this review have several limitations that impact the overall findings. It was commonly found that there was no baseline dietary intake analysis conducted. Four studies did conduct baseline dietary analysis, such as a food diary analysis^(48)^, *n*-3 blood analysis^(49)^, food waste and blood analysis (vitamin A, E, C, B_5_, B_12_, thiamine, riboflavin, niacin, pyridoxine, folates, Ca, Mg, Cu, Fe, Zn, Se, Mn and Cr)^(44)^ and blood analysis (vitamin A, B_5_, B_6_, E, thiamine, riboflavin, niacin, folic acid, Fe, Cr, Ca, Mn, Cu, Se, Zn and Mg)^(45)^. Identifying underlying nutritional deficiencies in each study population could provide a clearer picture of which nutritional issues may be causing poor outcomes.

Despite including various study types and locations, the lack of homogeneity among the included studies limited the ability to directly compare the effects of interventions on outcomes through a meta-analysis. A subgroup metanalysis was considered with the supplementation studies, as there were seven included in this review. Ultimately, this was decided against due to the heterogeneity between supplementation studies. It was difficult to identify the precise effects of supplements due to the variety of supplements, and the combination of supplement types used in the research included in this review. It is impossible to ascertain whether any improvements were due to any specific vitamin deficiencies being corrected, or if the offering of fatty acids, that is, *n*-3, were the cause, or if there was a synergistic effect of combined supplements.

Additionally, this review highlights the lack of females in research regarding prisoner populations.

Only one study focused solely on female participants^(43)^, with one other study including both females and males^(44)^. This reduces the generalisability of results across sexes, and therefore, future studies should consider the inclusion of females, particularly due to females having different nutritional needs from males^(77)^. Similarly, the majority of included studies recruited adult populations, with only four of the eleven studies included having recruited young offenders. As with females, the nutrition requirements differ across the life course, and this is something that should be considered.

### Future directions

The heterogeneity presented in the included studies, particularly among those using supplementation, creates difficulties in fully interpreting the results identified. A key theme that has emerged over the discourse of the literature reviewed is a lack of baseline dietary assessment. Future studies would benefit from conducting dietary analysis at baseline, to identify specific population group nutritional needs. This would help elucidate changes in participant nutrition and, in turn, enable research to identify the underlying mechanisms behind potential beneficial results.

Factors that have also limited the effects of interventions include the lack of stricter study criteria, which was noted in particular for aggression outcomes. Recruiting populations that exhibit high levels of aggressive behaviour at baseline would enable researchers to observe a greater change, compared with populations with low aggression at baseline. Similarly, the results of Johnson *et al.* ^(43)^ were impacted by a study population with high resilience, reducing the ability to identify improvements.

We would also encourage further research including female participants, as females were generally lacking in the included studies. The underrepresentation of females in the research is concerning particularly within the context of health and nutrition as females require different nutritional needs than males^(77)^. While we recognise males make up the majority of prisoners globally, and therefore, it is somewhat understandable females do still account for 6·9 %^(78)^. Additionally, there are various subgroups within prisons that were mostly absent from the included studies in this review, for example, pregnant women, those with chronic diseases, elderly prisoners and different ethnic groups.

Finally, as this was the first review of its kind, and due to the heterogeneity of the included studies, both overall and within the three intervention groups identified, a meta-analysis at this stage was felt to be of little benefit. However, a future review would benefit by narrowing in on one of the intervention types identified or conducting subgroup metanalysis for each intervention type once more emerging evidence is identified and published. This could allow for a meta-analysis to provide improved estimates of the effects seen and increase the generalisability of study results.

### Conclusion

Nutrition is fundamental to a person’s overall health and well-being, and nutritional interventions have been identified as a possible means to improve the prison environment, with the greatest effect seen in the reduction in the number of rule violations. However, this review identifies that the literature concerning prisoners, mental health and nutrition is still in its infancy and that the results discussed should be interpreted with caution. We encourage further research in this area, to fully understand the effects nutrition can have, and this review sets out future directions that could be taken, to contribute to the benefit of the prison population.

## Supporting information

Poulter et al. supplementary material Poulter et al. supplementary material

## Supplementary material

For supplementary material/s referred to in this article, please visit https://doi.org/10.1017/S0007114524000849.
