# Supplementary material for: A systematic review of the effect of dietary and nutritional interventions on the behaviours and mental health of prisoners
Source: Br J Nutr. 2024 Apr 29;132(1):77–90. doi: 10.1017/S0007114524000849 (PMC11420884; doi:10.1017/S0007114524000849)
Supplement: Poulter et al. supplementary material [file S0007114524000849sup001.docx]

Supplementary Material

Appendix 1: List of Search Terms.

|  | PubMed/Medline | Web of Science, EMBASE, PsycINFO, and CINAHL |
| --- | --- | --- |
| Population | (Confinement OR Detain* OR Detention OR Imprison* OR Incarcerate* OR "Inmate" OR Jail OR "Juvenile detention" OR Offender OR Prison* OR "Prisons"[Mesh] OR YOI OR "Youth detention" OR Offend* OR "Young Offender Instil*" OR HMYOI OR "Correctional Facilities"[Mesh] OR "Correctional facilities" OR Gaol) | (Confinement OR Detain* OR Correctional OR Detention OR HMYOI OR Imprison* OR Incarcerate* OR "Inmate" OR Jail OR Offend* OR Offender OR Prison* OR "Young Offender instil*" OR YOI "Correctional facilities" OR Gaol) |
| Intervention | ("Diet Change" OR "Dietary Change" OR "Diet therapy" OR "Diet Therapy"[Mesh] OR "Dietary intake" OR "Diet Intake" OR "Dietary Supplements" OR "Dietary Supplements"[Mesh] OR Menu OR "Mineral supplement*" OR "Multivitamin Supplement*" OR "Nutritional Support" OR "Nutrition Support" OR "Nutritional Support"[Mesh] OR "Nutrition Therapy" OR "Nutrition Therapy"[Mesh] OR "Vitamins"[Mesh] OR "Diet intervene*" OR "Dietary interven*" OR "Fatty acid" OR "Multivitamin Supplement*" OR "Vitamin-mineral supplement*" OR "Mineral supplement*" OR "Vitamin supplement*" OR "Diet supplement*" OR "Dietary supplement*" OR "Dietary Modification*" OR "Diet Modification*") | ("Diet Change" OR "Dietary Change" OR “Diet therapy” OR "Dietary intake" OR "Diet Intake" OR “Dietary Supplements” OR "Diet supplement*" OR Menu OR "Nutritional Support" OR "Nutrition Support" OR "Nutrition Therapy" OR "Nutritional Therapy" OR "Diet intervent*" OR “Dietary Interven*” OR "Diet Therap*" OR "Dietary Modification*" OR "Diet Modification*" OR “Fatty acid” OR "Multivitamin Supplement*" OR “Vitamin-mineral supplement*” OR “Mineral supplement*” OR “Vitamin supplement*” OR "Dietary supplement*") |
| Outcomes | (Aggres* OR Anxiety OR Antisocial OR Behavi* OR Depressi* OR "Depression"[Mesh] OR Emotional OR Happiness OR Mental OR "Mental Health" OR "Mental Health"[Mesh] OR "Self esteem" OR "Self-harm" OR "Self-Injurious Behavior"[Mesh] OR Suici* OR "Suicide Ideation" OR Welfare OR Well* OR Social OR "self-efficacy" OR "self-efficacy" OR "resilience" OR "empowerment" OR "social participation" OR "mental capital" OR Mental Disorders[Mesh] OR Mood) | (Aggres* OR Anxiety OR Antisocial OR Behavi* OR Depressi* OR Emotional OR Happiness OR Mental OR "Mental Health" OR Social OR "Self-esteem" OR "Self-harm" OR Suicid* OR "Suicide Ideation" OR Welfare OR Well* OR Nonsuicidal OR Mood OR “self-efficacy” OR “self-efficacy” OR “resilience” OR “empowerment” OR "Depressive symptoms" OR “social participation”) |
| Filter | English language only | English language only |

Appendix 2: General characteristics of excluded studies, and reasons for exclusion.

| **Ref.** | **Author, Year** | **Study Design** | **Group Studied** | **Exposure/Intervention** | **Outcome** | **Reason for Exclusion** |
| --- | --- | --- | --- | --- | --- | --- |
| [83] | Brown *et al*., 1990^(83)^ | Open trial pilot | Patients previously been convicted of driving under the influence, N=60 | Supplement, diet education | Self-reports on stress, depression, irritability, paranoia, anxiety, and drug craving. Along with reporting improvements in energy, self-confidence, and feelings of well-being | Not a population of interest |
| [84] | Faintuch *et al*., 2001^(84)^ | Retrospective, observational | Adult prisoners, N=8 | No intervention | Effect of refeeding procedures | Not a diet intervention |
| [85] | McKinnon *et al*., 2018^(85)^ | Cross-sectional | Inpatients at a secure facility, N=73 | No intervention | Vitamin D serum levels | Not a diet intervention |
| [86] | Rahman *et al*., 2017^(86)^ | Case-control | Male/female detention camp residents, N=61 | Thiamine IV, and oral supplement | Bilateral leg swelling | Not an outcome of interest |
| [87] | Shniderman & Solberg., 2015^(87)^ | Review | N/A | N/A | N/A | Review paper |
| [88] | Soomro *et al*., 2016^(88)^ | Epidemiological | Prisoners, N=300 | No intervention | Sociodemographic profile, eating and addictive habits, stress level, lifestyle, diseases, and height and weight | Not a diet intervention |
| [89] | Stanikowski *et al*., 2020^(89)^ | Cross-sectional | Diets from prisoners, N=30 | No intervention | Diet meeting recommended dietary allowance | Not a diet intervention |
| [90] | Starzomska *et al*., 2006^(90)^ | Review | N/A | N/A | N/A | Not a diet intervention |
| [91] | Virkkunen *et al*., 1987^(91)^ | Cross-sectional | Male offenders, N=34 | No intervention | Plasma phospholipid essential fatty acids and prostaglandins | Not a diet intervention |
| [92] | Virkkunen *et al*., 2007^(92)^ | Cross-sectional | Male offenders, N=96 | No intervention | Energy substrate metabolism | Not a diet intervention |
| [93] | Zaalberg., 2019^(93)^ | Review | N/A | N/A | N/A | Review paper |

Appendix 3: ROB 2 Risk of Bias Assessment for Randomised Studies.

| **Ref.** | **Author, Year** | **Randomisation Process** | **Deviations from Intended Interventions** | **Missing Outcome Data** | **Measurement of the Outcome** | **Selection of the Reported Results** | **Overall Bias** |
| --- | --- | --- | --- | --- | --- | --- | --- |
| [43] | Bachorowski *et al*., (1994)^(43)^ | Some concerns | Some concerns | Some concerns | Low | Low | Some concerns |
| [47] | Hansen *et al*., (2014)^(47)^ | Low | Some concerns | Low | Low | Low | Low |
| [48] | Hansen *et al*., (2015)^(48)^ | Low | Some concerns | Low | Low | Low | Low |
| [44] | Cortie *et al*., (2020)^(44)^ | Low | Low | Low | Some concerns | Low | Low |
| [46] | Gesch *et al*., (2002)^(46)^ | Low | Low | Low | Low | Low | Low |
| [50] | Raine *et al*., (2020)^(50)^ | Low | Low | Low | Low | Low | Low |
| [51] | Schoenthaler *et al*., (1997)^(51)^ | Low | Low | Low | Low | Low | Low |
| [52] | Schoenthaler *et al*., (2021)^(52)^ | Low | Low | Low | Low | Low | Low |
| [53] | Zaalberg *et al*., (2010)^(53)^ | Low | Low | Low | Low | Low | Low |

Appendix 4: ROBINS-I Risk of Bias Assessment for Non-Randomised Studies

| **Ref.** | **Author, Year** | **Confounding** | **Participant Selection** | **Intervention Classification** | **Deviations from Intended Interventions** | **Missing Data** | **Measurement of Outcomes** | **Selection of the Reported Results** | **Overall Bias** |
| --- | --- | --- | --- | --- | --- | --- | --- | --- | --- |
| [49] | Johnson *et al*., (2018)^(49)^ | Critical | Low | Low | Low | Low | Moderate | Low | Critical |
| [45] | D'Asaro *et al*., (1975)^(45)^ | Critical | Critical | Low | Low | Critical | Serious | Low | Critical |
